# STEMI, Revascularization, and Peak Troponin by Adverse Pregnancy Outcomes in Women With Myocardial Infarction

**DOI:** 10.1016/j.jacadv.2024.101088

**Published:** 2024-07-05

**Authors:** Moa Handmark, Annie Lin, Andreas Edsfeldt, Giovanna Sarno, Abigail Fraser, Janet W. Rich-Edwards, Isabel Gonҫalves, Mats Pihlsgård, Simon Timpka

**Affiliations:** a Perinatal and Cardiovascular Epidemiology and Lund University Diabetes Centre, Department of Clinical Sciences Malmö, Lund University, Malmö, Sweden; b Cardiovascular Research - Translational Studies, Lund University, Malmö, Sweden; c Wallenberg Center for Molecular Medicine, Lund University, Lund, Sweden; d Department of Cardiology, Skåne University Hospital, Lund/Malmö, Sweden; e Department of Medical Sciences, Cardiology and Uppsala Clinical Research Center, Uppsala University, Uppsala, Sweden; f Population Health Science, Bristol Medical School, University of Bristol, Bristol, UK; g Division of Women’s Health, Department of Medicine, Brigham and Women’s Hospital and Harvard Medical School, Boston, Massachusetts, USA; h Department of Obstetrics and Gynaecology, Skåne University Hospital, Malmö, Sweden

**Keywords:** myocardial infarction, pregnancy history, STEMI

## Abstract

**Background:**

Women with a history of adverse pregnancy outcomes have a higher risk of coronary heart disease. Emerging evidence suggests that women with a history of preeclampsia have a different pattern of overall coronary atherosclerosis and that they at the time of myocardial infarction (MI) more frequently present with ST-segment elevation MI (STEMI) compared to women with no such history.

**Objectives:**

The purpose of this study was to determine whether among women with MI, those with a history of adverse pregnancy outcomes are more likely to present with STEMI or other clinical characteristics indicating a more severe myocardial injury.

**Methods:**

The study sample consisted of 8,320 women aged ≤65 years with first MI in Sweden 2007 to 2022. Regression models were used to estimate the association between adverse pregnancy outcomes (hypertensive disorders of pregnancy [non-preeclamptic hypertension and preeclampsia], small for gestational age [SGA] infant, and preterm delivery) and STEMI, invasive revascularization, and high troponin, while considering known predictors of coronary heart disease.

**Results:**

In total, 3,128 (38%) of women suffered STEMI. The adjusted OR of presenting with STEMI were higher in women with a history of preterm preeclampsia (OR: 1.40; 95% CI: 1.05-1.88), or an SGA infant (OR: 1.30; 95% CI: 1.13-1.50) compared to women with no such history, as well as for in-hospital revascularization. Stratified by infarct type, troponin levels did not differ by adverse pregnancy outcome history.

**Conclusions:**

Among women with a first MI, a history of preterm preeclampsia or SGA infant were associated with STEMI and invasive revascularization.

Hospitalizations for myocardial infarction (MI) show an increasing trend in younger women.^1^ The link between adverse pregnancy outcomes, such as preeclampsia and preterm delivery, and incident coronary heart disease (CHD) has become evident during the last 2 decades.^2^, ^3^, ^4^, ^5^ More recently, we showed that a history of adverse pregnancy outcomes in middle age is associated with more advanced coronary artery atherosclerosis and a difference in overall distribution of coronary atherosclerosis as identified through coronary computed tomography angiography.^6^

Studies on the clinical characteristics of MI in women by adverse pregnancy outcome history have indicated that such a history is associated with an earlier presentation of MI following delivery,^7^ and that women with a history of preeclampsia present with a higher proportion of ST-segment elevation MI (STEMI) compared to women with no such history.^8^^,^^9^ As clinical characteristics of MIs are important parameters for estimating the acute myocardial damage, treatment, and prognosis,^10^ determining the infarct type, either STEMI or non-STEMI (NSTEMI), is a key aspect of the acute clinical decision-making. Other important clinical characteristics of MI include high peak troponin value, which is associated with infarct size and worse outcome in patients with acute MI.^11^^,^^12^ However, previous studies on the association between adverse pregnancy outcome history and clinical characteristics of MI are based on small sample sizes, which have limited analyses on the less prevalent but severe pregnancy complications such as preterm preeclampsia.^7^, ^8^, ^9^

This study aimed to investigate clinical indicators of a more severe myocardial injury at first MI by history of adverse pregnancy outcomes in a large national sample of parous women in Sweden. We hypothesized that a more severe myocardial injury, as reflected by STEMI, invasive revascularizations, and high peak troponin, would be more common in women with a history of each adverse pregnancy outcome than in women without such history.

## Methods

**Figure 1:**
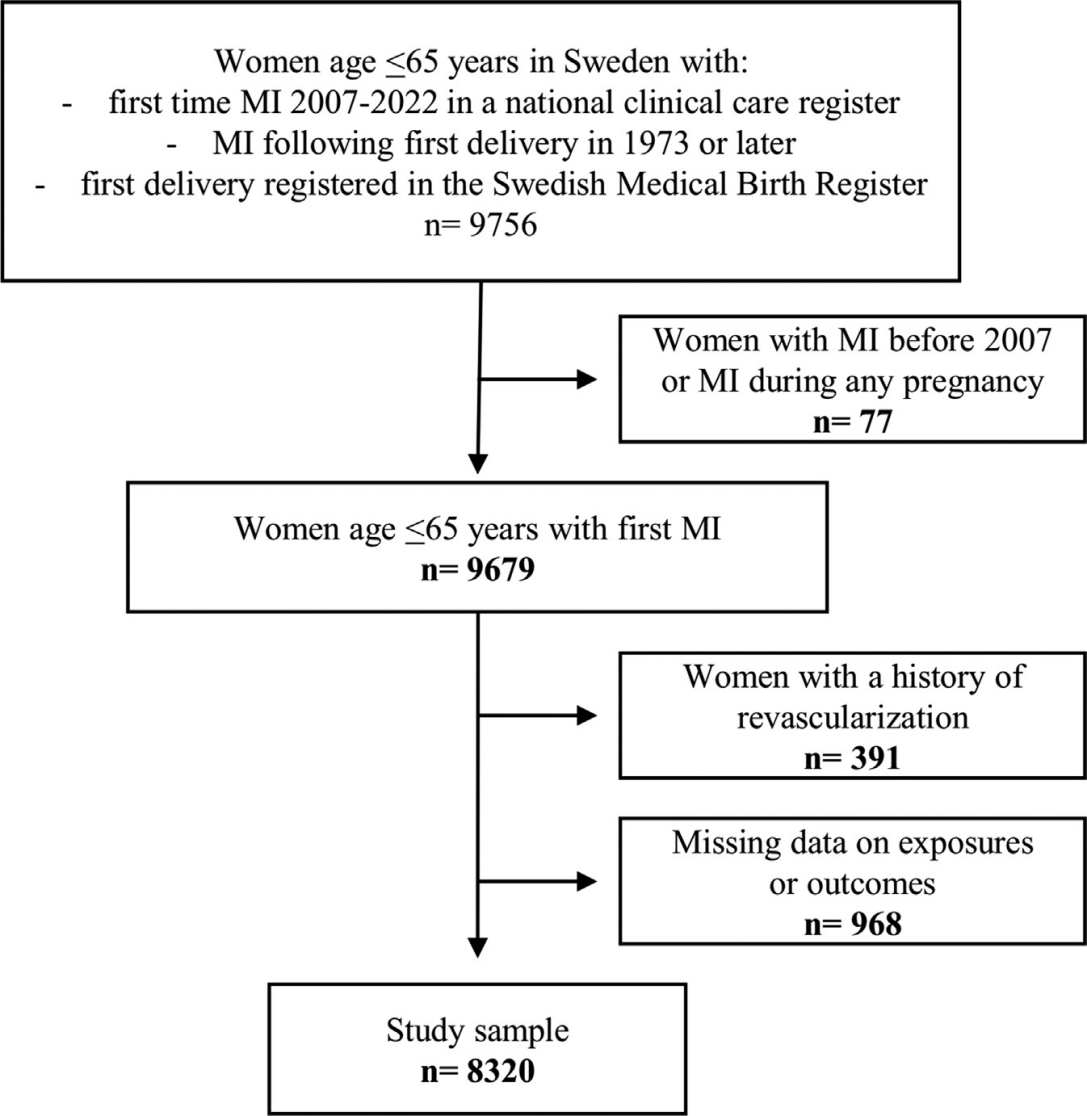
**Flowchart of Study Sample** Figure shows the inclusion and exclusion criteria for the study sample. MI = myocardial infarction.

For this study of women with a first MI in Sweden 2007 to 2022, the data primarily originated from 2 Swedish health care registers: Register of Information and Knowledge About Swedish Heart Intensive Care Admissions (RIKS-HIA) and the Swedish Medical Birth Register (MBR). Women were included if, after their first delivery, they were diagnosed with a first-time MI in 2007 to 2022 at age ≤65 years and had their first delivery recorded in the MBR (Figure 1). Only MIs in RIKS-HIA after 2007 were included as not all relevant variables in RIKS-HIA were routinely recorded up to that point. Women >65 years of age were not included to avoid differential inclusion of older women as a result of delivery data only being available from 1973 and onward and to harmonize the upper age limit with previous studies.^6^^,^^7^ To ensure that first-time MIs were captured, the national patient registry was used to exclude women with MIs occurring prior to 2006 (n = 73). Women with MI during any pregnancy (n = 4) were also excluded, as were women with prior revascularization procedures at the time of MI (n = 391). Lastly, women with missing data on adverse pregnancy outcome history or any of the outcomes (n = 968) were excluded. The register data were linked using the Swedish unique personal identity number.^13^ The study was approved by the Ethical Review Board in Lund (2015/792, 2018/23) and the Swedish Ethical Review Authority (2021-04863).

### Data on adverse pregnancy outcomes

Exposure data on adverse pregnancy outcomes originated from the MBR. The MBR is a comprehensive register that has collected data on almost all pregnancies leading to delivery in Sweden since 1973.^14^ Only women with their first delivery registered in the Swedish MBR were included in order to ascertain a more complete delivery history, and deliveries recorded after first MI were not included in the study. Hypertensive disorders of pregnancy were defined in accordance with International Classification of Diseases- 8, -9, and -10 (Supplemental Appendix), and coded as a binary variable. It was further categorized as preterm preeclampsia (delivery gestational week ≤36 + 6), term preeclampsia (delivery gestational week 37 + 0 or later), and non-preeclampsia hypertension (either essential hypertension in early pregnancy or gestational hypertension). For the purpose of this study, hypertensive disorders of pregnancy were defined based on a woman’s most severe diagnosis prior to her first MI. Preterm delivery was defined as delivery before 37 + 0 weeks of gestation and further defined as a woman’s most preterm delivery prior to first MI. For subgroup analyses, preterm delivery was divided into very preterm delivery (22 + 0-33 + 6 weeks of gestation) and late preterm delivery (34 + 0-36 + 6 weeks of gestation). Ever small for gestational age (SGA) was defined as ever delivering an infant >2 standard deviations below the normal weight by infant sex and length of pregnancy.^15^

### Data on first time myocardial infarction

Outcome data on first time MI were collected from RIKS-HIA, a Swedish national quality register collecting data on patients admitted to cardiac care in Sweden.^16^ RIKS-HIA’s standardized criteria for MI used by all participating hospitals have been previously described.^17^ MI was defined using International Classification of Diseases-10 codes corresponding to acute MI diagnosis (I21).

STEMI (vs NSTEMI), invasive revascularization procedure, and peak troponin were used as indicators of a more severe myocardial injury. Firstly, the association between STEMI (vs NSTEMI) at the time of first MI and a history of adverse pregnancy outcome was studied. STEMI is the most severe form of presentation of acute coronary syndrome, traditionally associated with transmural infarction.^18^ Compared with NSTEMI, STEMI is associated with larger myocardial injury and worse short-term prognosis.^19^^,^^20^ In RIKS-HIA, infarct type is defined by the treating physician in each individual case and then recorded in the registry as STEMI or NSTEMI. Secondly, the association between an invasive revascularization procedure at the time of first MI and a history of adverse pregnancy outcome was studied. Invasive treatment is routinely performed in a STEMI setting, guidelines recommending invasive treatment within 120 minutes from diagnosis.^21^ In the case of NSTEMI patients, guidelines recommend an invasive strategy to all patients and revascularization if needed. In this study, invasive revascularization procedure was defined as percutaneous coronary intervention or coronary artery by-pass surgery during the hospital stay corresponding with first MI diagnosis. Third and lastly, as high peak troponin value is a predictor of infarct size and worse outcome following MI,^11^^,^^12^ peak troponin value, as recorded in the register, was used to study the association between adverse pregnancy outcome history and infarct size. To study myocardial injury based on troponin release, each type of peak troponin used in the study sample (troponin T [n = 676], highly sensitive troponin T [n = 5,110], troponin I [n = 1,597], and highly sensitive troponin I [n = 937]) was log transformed as the troponin data are highly skewed. To harmonize the different types of troponin data and allow for a comprehensive peak troponin value analysis, z-scores were separately calculated for each type of troponin. This transformation allowed for a pooled analysis on myocardial injury, based on peak troponin, irrespective of troponin type.

Age was calculated from the woman’s year of birth and the discharge year of her first-time MI. Body mass index (BMI) at MI was calculated as weight in kilograms divided by height in meters squared (kg/m^2^) and included as a continuous variable. BMI <14 or >55 kg/m^2^ was set to missing. In this study, diabetes was defined as having a known diagnosis of diabetes or receiving treatment for diabetes at the time of MI, whereas hypertension is defined by RIKS-HIA as receiving antihypertensive drugs at the time of MI. RIKS-HIA defines smoking as never-smoker, current smoker, or ex-smoker (>1 month). For the purpose of this study, treatment for dyslipidemia was defined as receiving lipid-lowering agents at the time of MI.

### Statistical analysis

Characteristics of the study sample are presented as means or percentages. Logistic regression was used to assess the association between adverse pregnancy outcome history and STEMI at first MI. Model I includes adverse pregnancy outcome history (hypertensive disorder of pregnancy, preterm delivery, or SGA infant) and age. Model II additionally includes BMI, diabetes, hypertension, smoking status, and treatment for dyslipidemia as established predictors for CHD. Similarly, adverse pregnancy outcome history and invasive revascularization at the time of first MI was studied as described above for the STEMI analysis. To study the mean difference in peak troponin by adverse pregnancy history, a linear regression model including covariables as described above for the STEMI analysis was used. However, to understand the extent to which adverse pregnancy outcome history was associated with particularly high peak troponin, that is, assuming a nonlinear association, the association between adverse pregnancy outcome history and particularly high troponin value indicating a more severe myocardial injury was studied. Peak troponin ≥ the top quartile was used to indicate a particularly severe myocardial damage. This is a cutoff used before in studies analyzing biomarkers in cardiovascular research.^22^^,^^23^ Troponin analyses were also stratified by STEMI or NSTEMI as infarct type is associated with severity of myocardial injury.^18^^,^^20^

As a woman’s risk of ever experiencing an adverse pregnancy outcome rises with her total number of deliveries and parity is also known to be associated with future maternal cardiovascular disease,^24^ analyses additionally adjusting for parity in all models were performed. Analyses where preterm delivery was subcategorized into normotensive preterm delivery (no history of hypertension during pregnancy) and hypertensive preterm delivery were also performed, as preterm delivery is strongly associated with hypertensive disorders of pregnancy. Lastly, to assess the covariables effect on the model, analyses adjusting for each covariable separately in addition to age at MI were performed.

A total of 512 (6.2%) participants had missing data on at least 1 covariable and multiple imputation was used to impute missing values of these covariables. Twenty imputed data sets were created using multiple imputation by chained equations and data were analyzed with the command mi estimate in Stata. All major analyses were repeated using a complete case data set in which individuals with missing data (n = 512) were excluded. Model assumptions for the ordinary regression analysis were assessed by visual inspection of the QQ-plot of regression residuals and residuals vs predicted mean levels. For logistic models, residual deviance per degree of freedom was below 1 indicating a reasonable overall fit and no over-dispersion. The adequacy of chosen predictors and link function (logistic) were assessed through tests based on cumulative residuals. A significance level of *P* < 0.05 was used for hypothesis testing. The statistical analysis was conducted using Stata 16.0 (StataCorp LLC).

## Results

Table 1 shows the characteristics of the study sample by adverse pregnancy outcome history. Women with a history of any hypertensive disorders of pregnancy suffered MIs at a younger age and were more likely to have diabetes, hypertension, treatment for dyslipidemia as well as a higher BMI at the time of MI. When dividing hypertensive disorders of pregnancy into subgroups, women with a history of preterm preeclampsia were younger and more often had diabetes compared to women with a history of any hypertensive disorder of pregnancy. Women with a history of preterm delivery presented with MI at a younger age and more frequently with diabetes, hypertension, and/or treatment for dyslipidemia compared to women without a history of preterm delivery. Women with a history of an SGA infant were more often active smokers at the time of MI compared to women with no history of delivering an SGA infant.

**Table 1:** Patient Characteristics at First Myocardial Infarction by Adverse Pregnancy Outcome History (N = 8,320)

	Preterm Delivery		Small for Gestational Age Infant		Hypertensive Disorder of Pregnancy					Missing n (%)
	Ever PTD (n = 1,301)	No PTD (n = 7,019)	Ever SGA (n = 887)	No SGA (n = 7,433)	Ever HDP (n = 1,078)	Preterm PE (n = 206)	Term PE (n = 518)	Non-PE Hypertension (n = 354)	Normotensive (n = 7,242)	
Age, y (SD)	55.0 ± 7.2	56.3 ± 6.8	56.5 ± 6.8	56.1 ± 6.9	54.8 ± 7.3	52.4 ± 7.8	55.0 ± 7.0	56.1 ± 7.1	56.3 ± 6.8	-
Diabetes	296 (22.8)	1,047 (14.9)	133 (15.0)	1,210 (16.3)	262 (24.3)	68 (33.0)	109 (21.0)	85 (24.0)	1,081 (14.9)	28 (0.3)
Hypertension	545 (41.9)	2,683 (38.2)	371 (41.8)	2,857 (38.4)	625 (58.0)	114 (55.3)	296 (57.1)	215 (60.7)	2,603 (35.9)	45 (0.5)
Treatment for dyslipidemia	206 (15.8)	902 (12.8)	130 (14.7)	978 (13.2)	205 (19.0)	40 (19.4)	97 (18.7)	68 (19.2)	903 (12.5)	9 (0.1)
BMI, kg/m^2^	28.0 ± 5.8	27.9 ± 5.6	27.2 ± 5.4	28.0 ± 5.6	29.4 ± 5.9	29.1 ± 6.1	29.4 ± 5.9	29.7 ± 5.7	27.7 ± 5.5	305 3.7
Smoking										179 (2.2)
Never	367 (28.2)	2,124 (30.3)	175 (19.7)	2,316 (31.1)	424 (39.3)	86 (41.7)	205 (39.6)	133 (37.6)	2,067 (28.5)	
Ex-smoker	322 (24.7)	1,783 (25.4)	213 (24.0)	1,892 (25.4)	274 (25.4)	51 (24.8)	127 (24.5)	96 (27.1)	1,831 (25.3)	
Smoker	582 (44.7)	2,963 (42.2)	481 (54.2)	3,064 (41.2)	353 (32.7)	59 (28.6)	178 (34.3)	116 (32.8)	3,192 (44.1)	

Values are mean ± SD or n (%). PTD is defined as a woman’s most preterm delivery prior to her first myocardial infarction. HDP is defined as a woman’s most serious diagnosis prior to her first myocardial infarction.

BMI = body mass index; HDP = hypertensive disorder of pregnancy; PE = preeclampsia; PTD = preterm delivery; SD = standard deviation; SGA = small for gestational age.

### Myocardial infarction subtype by adverse pregnancy outcome history

In total, 3,128 (38%) women presented with STEMI and 5,192 (62%) women presented with NSTEMI. A history of preterm preeclampsia was associated with STEMI at the time of first MI in the fully adjusted model (OR: 1.40; 95% CI: 1.05-1.87), as was a history of a SGA infant (OR: 1.30; 95% CI: 1.13-1.50) (Table 2). Neither history of term preeclampsia nor non-preeclampsia hypertension was associated with STEMI. Preterm delivery was also not associated with STEMI. To determine whether any association between SGA infant and STEMI at the time of first MI may be driven by a history of hypertensive disorders of pregnancy, a secondary analysis restricted to women without a history of hypertensive disorders of pregnancy was performed. The association remained even in women without a history of hypertensive disorder of pregnancy (Supplemental Table 1).

**Table 2:** Association Between Adverse Pregnancy Outcome History and STEMI Among Women Presenting With First Myocardial Infarction (N = 8,320)

	Model I		Model II	
	OR (95% CI)	*P* Value	OR (95% CI)	*P* Value
Preterm delivery (n STEMI/n MI)				
Never preterm delivery (2,644/7,019)	1.00 (reference)		1.00 (reference)	
Ever preterm delivery (484/1,301)	0.99 (0.87-1.12)	0.84	0.98 (0.87-1.11)	0.76
Late preterm delivery (317/863)	0.97 (0.83-1.12)	0.64	0.96 (0.83-1.12)	0.60
Very preterm delivery (167/438)	1.03 (0.85-1.26)	0.76	1.02 (0.83-1.25)	0.83
Small for gestational age infant (n STEMI/n MI)				
Never small for gestational age infant (2,730/7,433)	1.00 (reference)		1.00 (reference)	
Ever small for gestational age infant (398/887)	1.40 (1.22-1.61)	<0.001	1.30 (1.13-1.50)	<0.001
Hypertensive disorder of pregnancy (n STEMI/n MI)				
Normotensive (2,735/7,242)	1.00 (reference)		1.00 (reference)	
Hypertensive disorder of pregnancy (393/1,078)	0.95 (0.83-1.09)	0.48	1.07 (0.94-1.23)	0.31
Preterm preeclampsia (85/206)	1.18 (0.89-1.57)	0.24	1.40 (1.05-1.87)	0.02
Term preeclampsia (179/518)	0.88 (0.73-1.06)	0.17	0.98 (0.81-1.18)	0.81
Non-preeclampsia hypertension (129/354)	0.95 (0.76-1.18)	0.63	1.06 (0.85-1.33)	0.61

MI = myocardial infarction; STEMI = ST-segment elevation myocardial infarction.

### Invasive revascularization procedure by adverse pregnancy outcome history

Women with a history of preterm preeclampsia were more likely to undergo invasive revascularization at the time of MI than women with no history of hypertensive pregnancy (fully adjusted OR: 1.43; 95% CI: 1.07-1.92). A history of delivering an SGA infant was also associated with invasive revascularization (OR: 1.20; 95% CI: 1.04-1.39). A history of any hypertensive disorder of pregnancy or term preeclampsia was not associated with invasive revascularization at the time of MI, nor was a history of preterm delivery (Table 3).

**Table 3:** Association Between Adverse Pregnancy Outcome History and Invasive Revascularization Procedure Among Women Presenting With First Time Myocardial Infarction (N = 8,320)

	Model I		Model II	
	OR (95% CI)	*P* Value	OR (95% CI)	*P* Value
Preterm delivery (n revascularization/n MI)				
Never preterm delivery (2,344/7,019)	1.00 (reference)		1.00 (reference)	
Ever preterm delivery (436/1,301)	1.01 (0.89-1.15)	0.82	1.01 (0.89-1.15)	0.91
Late preterm delivery (286/863)	0.99 (0.86-1.16)	0.95	0.99 (0.85-1.15)	0.88
Very preterm delivery (150/438)	1.05 (0.86-1.29)	0.61	1.05 (0.85-1.29)	0.66
Small for gestational age infant (n revascularization/n MI)				
Never small for gestational age infant (2,436/7,433)	1.00 (reference)		1.00 (reference)	
Ever small for gestational age infant (344/887)	1.30 (1.12-1.50)	<0.001	1.20 (1.04-1.39)	0.01
Hypertensive disorder of pregnancy (n revascularization/n MI)				
Normotensive (2,441/7,242)	1.00 (reference)		1.00 (reference)	
Hypertensive disorder of pregnancy (339/1,078)	0.91 (0.79-1.05)	0.19	1.03 (0.89-1.19)	0.69
Preterm preeclampsia (77/206)	1.21 (0.90-1.61)	0.20	1.43 (1.07-1.92)	0.02
Term preeclampsia (149/518)	0.80 (0.66-0.98)	0.03	0.89 (0.73-1.09)	0.27
Non-preeclampsia hypertension (113/354)	0.92 (0.73-1.16)	0.50	1.04 (0.82-1.31)	0.76

Results from logistic regression multiple imputation analysis.

Preterm delivery is defined as a woman’s most preterm delivery prior to her first MI. Hypertensive disorders of pregnancy are defined as a woman’s most serious diagnosis prior to her first MI.

Model I includes adverse pregnancy outcome history; age at first time MI [continuous].

Model II additionally includes diabetes [yes/no]; hypertension [yes/no]; treatment for dyslipidemia [yes/no]; smoking status [never smoker, current smoker, ex-smoker >1 month]; BMI [continuous].

BMI = body mass index; MI = myocardial infarction.

### High troponin value and infarct size by adverse pregnancy outcome history

No association was found between adverse pregnancy outcome history and log z-score troponin overall (Table 4). However, a history of an SGA infant was associated with a particularly high troponin value at the time of first MI in the fully adjusted model (OR: 1.18; 95% CI: 1.01-1.38) (Table 5). When stratifying by STEMI or NSTEMI, no association was found between troponin levels and any adverse pregnancy outcome history (Supplemental Tables 2 to 5).

**Table 4:** Association Between Adverse Pregnancy Outcome History and Log Z-Score Troponin Among Women Presenting With First Time Myocardial Infarction (N = 8,320)

	Model I		Model II	
	β (95% CI)	*P* Value	β (95% CI)	*P* Value
Preterm delivery				
Never preterm delivery	1.00 (reference)		1.00 (reference)	
Ever preterm delivery	0.001 (−0.06 to 0.06)	0.98	−0.001 (−0.06 to 0.06)	0.97
Late preterm delivery	−0.02 (−0.09 to 0.05)	0.61	−0.02 (−0.09 to 0.05)	0.55
Very preterm delivery	0.04 (−0.06 to 0.14)	0.42	0.04 (−0.06 to 0.14)	0.43
Small for gestational age infant				
Never small for gestational age infant	1.00 (reference)		1.00 (reference)	
Ever small for gestational age infant	0.07 (−0.005 to 0.14)	0.07	0.05 (−0.02 to 0.12)	0.19
Hypertensive disorder of pregnancy				
Normotensive	1.00 (reference)		1.00 (reference)	
Hypertensive disorder of pregnancy	0.02 (−0.05 to 0.08)	0.56	0.06 (−0.01 to 0.13)	0.07
Preterm preeclampsia	0.05 (−0.09 to 0.19)	0.48	0.10 (−0.04 to 0.24)	0.17
Term preeclampsia	0.05 (−0.04 to 0.14)	0.30	0.09 (−0.003 to 0.18)	0.06
Non-preeclampsia hypertension	−0.04 (−0.15 to 0.07)	0.45	−0.001 (−0.11 to 0.11)	0.99

Results from linear regression multiple imputation analysis.

Preterm delivery is defined as a woman’s most preterm delivery prior to her first MI. Hypertensive disorders of pregnancy are defined as a woman’s most serious diagnosis prior to her first MI.

Model I includes adverse pregnancy outcome history; age at first time MI [continuous].

Model II additionally includes diabetes [yes/no]; hypertension [yes/no]; treatment for dyslipidemia [yes/no]; smoking status [never smoker, current smoker, ex-smoker >1 month]; BMI [continuous].

BMI = body mass index.

**Table 5:** Association Between Adverse Pregnancy Outcome History and High Troponin (Within the Fourth Quartile) Value Among Women Presenting With First Time Myocardial Infarction (N = 8,320)

	Model I		Model II	
	OR (95% CI)	*P* Value	OR (95% CI)	*P* Value
Preterm delivery (n high troponin/n MI)				
Never preterm delivery (1,872/7,019)	1.00 (reference)		1.00 (reference)	
Ever preterm delivery (336/1,301)	0.97 (0.85-1.11)	0.70	0.97 (0.85-1.11)	0.67
Late preterm delivery (215/863)	0.92 (0.78-1.09)	0.34	0.92 (0.78-1.08)	0.31
Very preterm delivery (121/438)	1.08 (0.87-1.34)	0.49	1.08 (0.87-1.34)	0.49
Small for gestational age infant (n high troponin/n MI)				
Never small for gestational age infant (1,939/7,433)	1.00 (reference)		1.00 (reference)	
Ever small for gestational age infant (269/887)	1.23 (1.05-1.43)	0.01	1.18 (1.01-1.38)	0.03
Hypertensive disorder of pregnancy (n high troponin/n MI)				
Normotensive (1,920/7,242)	1.00 (reference)		1.00 (reference)	
Hypertensive disorder of pregnancy (288/1,078)	1.03 (0.89-1.19)	0.68	1.11 (0.96-1.29)	0.16
Preterm preeclampsia (60/206)	1.20 (0.89-1.63)	0.24	1.33 (0.98-1.82)	0.07
Term preeclampsia (141/518)	1.06 (0.86-1.29)	0.59	1.13 (0.92-1.39)	0.23
Non-preeclampsia hypertension (87/354)	0.91 (0.71-1.16)	0.44	0.97 (0.76-1.25)	0.82

Results from logistic regression multiple imputation analysis.

Preterm delivery is defined as a woman’s most preterm delivery prior to her first MI. Hypertensive disorders of pregnancy are defined as a woman’s most serious diagnosis prior to her first MI.

Model I includes adverse pregnancy outcome history; age at MI [continuous].

Model II additionally includes diabetes [yes/no]; hypertension [yes/no]; treatment for dyslipidemia [yes/no]; smoking status [never smoker, current smoker, ex-smoker >1 month]; BMI [continuous].

Abbreviations as in Table 3.

### Additional analyses

When additionally adjusting for parity in model I, the estimates did not notably change (data not shown). No association was found between normotensive preterm delivery and outcomes; hypertensive preterm delivery appeared to be elevated, consistent with the observation in Table 2 that preterm preeclampsia was associated with STEMI (Supplemental Tables 6 to 9). Supplemental Table 10 highlights the effect smoking status has on the association between preterm preeclampsia and STEMI. Preterm preeclampsia was associated with STEMI (OR: 1.35; 95% CI: 1.02-1.81) when adjusting for smoking status in addition to age at MI in a separate step. This is reflected in Table 1, which shows that women with a history of preterm preeclampsia were less likely to be active smokers than normotensive women at the time of MI. Supplemental Table 11 shows a similar analysis for invasive revascularization by hypertensive disorders of pregnancy history.

## Discussion

In women ≤65 years of age presenting with MI, a history of preterm preeclampsia and a history of an SGA infant were associated with clinical characteristics indicating a more severe myocardial injury (Central Illustration). In contrast, no evidence was found that a history of normotensive preterm delivery, term preeclampsia, or any history of hypertensive disorders of pregnancy were associated with any of the studied outcomes.

**Central Illustration:**
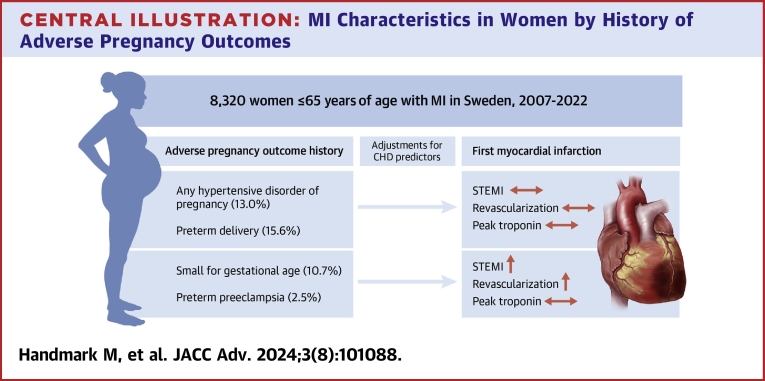
**MI Characteristics in Women by History of Adverse Pregnancy Outcomes** Among women ≤65 years of age with a first MI, a history of preterm preeclampsia or a history of an SGA infant were associated with STEMI and invasive revascularization. CHD = coronary heart disease; MI = myocardial infarction; SGA = small for gestational age; STEMI = ST-segment elevation myocardial infarction.

### Adverse pregnancy outcomes as markers of a more severe myocardial injury

The results indicate that women with a history of preterm preeclampsia and women with a history of an SGA infant present with more severe myocardial damage at the time of first MI. A history of delivering an SGA infant and a history of preeclampsia are thought to share underlying pathophysiological pathways, through endothelial dysfunction, for the development of future maternal CHD.^25^^,^^26^ Preeclampsia is usually categorized according to gestational length into preterm preeclampsia and term preeclampsia, where preterm preeclampsia is associated with more severe disease compared to term preeclampsia.^27^ In this study, some discordance was observed in outcome associations according to preeclampsia type, with no associations found for term preeclampsia. We have previously reported that a history of term preeclampsia, but not preterm preeclampsia, is associated with a lower risk of restenosis following percutaneous coronary intervention.^28^ Taken together, these results indicate a heterogeneity by type of preeclampsia and the association with presentation and outcome of future CHD in women.

STEMI typically arises from a thrombotic occlusion of a coronary artery,^18^ and the lesion localization is known to be associated with patient outcome. In general, proximal coronary artery disease and coronary artery disease located in specific vessels are known factors to be associated with worse prognosis after MI.^29^^,^^30^ In a recent study where we studied coronary computed tomography findings by adverse pregnancy outcome history (preeclampsia, gestational hypertension, gestational diabetes, SGA, and preterm delivery) in middle-aged women, we found adverse pregnancy outcomes to be associated with coronary artery atherosclerosis.^6^ We also found a history of preeclampsia, gestational hypertension, and SGA to be associated with a difference in coronary atherosclerosis segment distribution compared to women with no history of adverse pregnancy outcomes. While these previous findings are of unknown clinical relevance, they might help to understand the difference in MI presentation reported in this study. An association with clinical characteristics such as STEMI could reflect a difference in CHD development in women with a history of preterm preeclampsia or a history of SGA infant, compared to women with no such history.

The results presented in this study add to the results presented by Grand’Maison et al^8^ and McDonald et al ^9^ from smaller studies where among women with MI, women with a history of preeclampsia had a higher risk of presenting with STEMI compared to women with normotensive pregnancies. They also expand on the recent results presented by Countouris et al,^7^ where no associations between adverse pregnancy outcomes and clinical characteristics indicating a more severe myocardial injury were shown. The study by Countouris et al did not include data on history of delivering an SGA infant and preeclampsia was not analyzed by timing of delivery.

### Preterm delivery and future maternal coronary heart disease

Women with a history of preterm delivery presented with similar clinical MI characteristics as other parous women at the time of MI, though presenting at a younger age compared to other women. The underlying mechanisms of the association between preterm delivery and future maternal cardiovascular disease/CHD are not yet fully understood. Previous studies have shown that the trajectories of established cardiovascular risk factors of women with preterm delivery history are similar throughout adulthood to those of other women^31^ and that the traditional cardiovascular risk factors explain only a minor part of the increased risk of cardiovascular disease associated with preterm delivery.^5^^,^^32^ The risk of future CHD in women with a history of preterm delivery could therefore be partially driven by other, unknown factors, and these women might present differently with coronary events—and have a different prognosis—compared to parous women without a history of preterm delivery. We have previously shown that women with a history of preterm delivery have a worse prognosis in a secondary prevention setting, having a higher risk of adverse outcomes after coronary artery stenting.^33^ In the previously mentioned study on coronary computed tomography angiography findings by adverse pregnancy outcome history, we found women with a history of preterm delivery had a higher coronary artery calcium score compared to women without a history of adverse pregnancy outcomes,^6^ indicating a higher risk of future coronary events.

### Strengths and limitations

The main strength of this study is a large comprehensive national sample of women with first time MI, originating from data collected over decades in established, well-known, and well-curated registers.^34^^,^^35^ As such, the sample size is considerably larger than hitherto published studies in the area.

This study also had some limitations. Women with first time MI prior to 2007 were excluded in the study as not all variables included in this study were routinely collected until then, and women >65 years of age were excluded as inclusion of older women was limited by the lack of delivery data prior to 1973. However, it should be noted that the association between adverse pregnancy outcomes and future CHD has been shown to be more prominent in younger age groups.^36^ Pregnancies from the 70s and early 80s are at risk of being misclassified regarding pregnancy dating, as pregnancy dating with obstetric ultrasound was not used clinically until the 70s in Sweden. In addition, it should be noted that patient transfers between hospitals and wards present a risk of peak troponin values being inaccurately recorded in a minority of cases. However, both misclassifications related to pregnancy dating and troponin values would at most attenuate any associations. Furthermore, model II is adjusted for CHD predictors between the delivery and MI; while this could be considered over-adjustment for factors that might mediate the association between adverse pregnancy outcomes and future CHD, these post-pregnancy factors are also likely markers for risk factor status at the time of the pregnancy, for which we lack data. For example, adjustment for smoking could be an example of controlling for smoking at the time of the pregnancy. As data on MI mechanism were not available, we have not studied the proportion of MIs due to spontaneous coronary artery dissection or other nonatherosclerotic MIs. Lastly, it should also be noted that before generalizing our results to other populations, the relative ethnic homogeneity of the study sample should be considered.

## Conclusions

Among women ≤65 years of age presenting with a first MI, a history of preterm preeclampsia and of SGA infant were associated with STEMI and invasive revascularization. This adds to the evidence base linking adverse pregnancy outcomes and maternal cardiovascular risk by demonstrating that history of preterm preeclampsia and SGA are also linked to MI severity. The extent to which these findings are explained by divergent CHD development among these subgroups of women warrants further study.

Perspectives **COMPETENCY IN MEDICAL KNOWLEDGE:** Adverse pregnancy complications are sex-specific risk factors for future coronary heart disease and coronary heart disease is the leading cause of mortality for women world-wide. A history of adverse pregnancy outcomes might be a marker of a more severe myocardial injury at the time of myocardial infarction.

**TRANSLATIONAL OUTLOOK 1:** Further studies on adverse pregnancy outcomes and primary prevention for coronary heart disease would expand further on the knowledge of adverse pregnancy outcomes and future coronary heart disease.

**TRANSLATIONAL OUTLOOK 2:** The etiological mechanism by which a history of preterm preeclampsia and/or delivering a small for gestational infant is linked to myocardial infarction severity at the time of diagnosis needs to be further studied.

## Funding support and author disclosures

This work was supported by grants awarded to Dr Timpka from the 10.13039/501100004359Swedish Research Council (2019-02082), The 10.13039/501100003793Swedish Heart-Lung Foundation (20180312), Public research support via the Faculty of Medicine at 10.13039/501100003252Lund University (ALF: YF-ALF, ALF project), The 10.13039/501100007687Swedish Society of Medicine (SLS-885331), The Jeansson Foundations, Stockholm, Sweden, and Åke Wiberg Foundation, Stockholm, Sweden. Dr Gonҫalves received grants from the 10.13039/501100004359Swedish Research Council, the Swedish Heart and Lung Foundation, 10.13039/501100011077Skåne University Hospital funds and Lund University Diabetes Center (10.13039/501100004359Swedish Research Council - Strategic Research Area Exodiab Dnr 2009-1039, Linnaeus grant Dnr 349-2006-23 and the 10.13039/501100001729Swedish Foundation for Strategic Research Dnr IRC15-006). All other authors have reported that they have no relationships relevant to the contents of this paper to disclose.
