# Supplementary material for: STEMI, Revascularization, and Peak Troponin by Adverse Pregnancy Outcomes in Women With Myocardial Infarction
Source: JACC Adv. 2024 Jul 5;3(8):101088. doi: 10.1016/j.jacadv.2024.101088 (PMC11277779; doi:10.1016/j.jacadv.2024.101088)
Supplement: Supplementary data [file mmc1.docx]

**Supplemental Appendix**

# **ICD codes for hypertensive disorders of pregnancy**

ICD 8 used during the years 1968-1986

ICD 9 used during the years 1987-1996

ICD 10 used from 1997 and forward.

Depending on year of diagnosis we defined preeclampsia as 637.03, 637.04 and 637.09 when using ICD 8; 642E and 642F when using ICD 9; 014.0, 014.1, 014.1A, 014.1B and 014.9 when using ICD 10. Gestational hypertension was defined as 637.01 when using ICD 8, 642D or 642X when using ICD 9, 013.9 when using ICD 10.

| **Supplemental Table 1.** Association between history of small for gestational age infant and STEMI in women presenting with first time myocardial infarction, no history of hypertensive disorder of pregnancy (n=7242) | | | | |
| --- | --- | --- | --- | --- |
|  | **Model I** | | **Model II** | |
|  | OR (95% CI) | *p* | OR (95% CI) | *p* |
| **Small for gestational age infant**  (n STEMI / n MI) |  |  |  |  |
| Never small for gestational age infant  (2,421 / 6564) | 1 (reference) |  | 1 (reference) |  |
| Ever small for gestational age infant  (314 / 678) | 1.47 (1.25;1.72) | <0.001 | 1.34 (1.13;1.57) | 0.001 |
|  |  |  |  |  |
| Results from logistic regression multiple imputation analysis.  Model I includes adverse pregnancy outcome history; age at first time MI [continuous]  Model II additionally includes diabetes [yes/no]; hypertension [yes/no]; treatment for dyslipidaemia [yes/no]; smoking status [never smoker, current smoker, ex-smoker >1 month]; BMI [continuous].  * BMI: body mass index; CI: confidence interval; MI: myocardial infarction; OR: odds ratio. | | | | |

| **Supplemental Table 2.** Association between adverse pregnancy outcome history and log z-score troponin among women presenting with first time myocardial infarction, only STEMI cases (n=3128) | | | | |
| --- | --- | --- | --- | --- |
|  | **Model I** | | **Model II** | |
|  | β (95% CI) | *p* | β (95% CI) | *p* |
| **Preterm delivery** |  |  |  |  |
| Never preterm delivery | 1 (reference) |  | 1 (reference) |  |
| Ever preterm delivery | -0.05 (-0.14;0.04) | 0.30 | -0.05 (-0.14;0.04) | 0.30 |
| Late preterm delivery | -0.06 (-0.17;0.05) | 0.32 | -0.06 (-0.17;0.05) | 0.30 |
| Very preterm delivery | -0.03 (-0.18;0.12) | 0.65 | -0.03 (-0.18;0.12) | 0.67 |
| **Small for gestational age infant** |  |  |  |  |
| Never small for gestational age infant | 1 (reference) |  | 1 (reference) |  |
| Ever small for gestational age infant | -0.03 (-0.13;0.07) | 0.58 | -0.03 (-0.13;0.07) | 0.58 |
| **Hypertensive disorder of pregnancy** |  |  |  |  |
| Normotensive | 1 (reference) |  | 1 (reference) |  |
| Hypertensive disorder of pregnancy | 0.01 (-0.09;0.12) | 0.78 | 0.02 (-0.08;0.13) | 0.67 |
| Preterm preeclampsia | -0.10 (-0.31;0.11) | 0.34 | -0.09 (-0.30;0.12) | 0.39 |
| Term preeclampsia | 0.12 (-0.03;0.26) | 0.12 | 0.12 (-0.02;0.27) | 0.10 |
| Non- preeclampsia  hypertension | -0.05 (-0.22;0.12) | 0.56 | -0.04 (-0.21;0.13) | 0.63 |
| Results from linear regression multiple imputation analysis. Only STEMI-cases.  Model I includes adverse pregnancy outcome history; age at first time MI [continuous]  Model II additionally includes diabetes [yes/no]; hypertension [yes/no]; treatment for dyslipidaemia [yes/no]; smoking status [never smoker, current smoker, ex-smoker >1 month]; BMI [continuous].  * BMI: body mass index; CI: confidence interval; MI: myocardial infarction; OR: odds ratio; STEMI: ST-elevation myocardial infarction. | | | | |

| **Supplemental Table 3.** Association between adverse pregnancy outcome history and log z-score troponin among women presenting with first time myocardial infarction, only NSTEMI cases (n=5192) | | | | |
| --- | --- | --- | --- | --- |
|  | **Model I** | | **Model II** | |
|  | β (95% CI) | *p* | β (95% CI) | *p* |
| **Preterm delivery** |  |  |  |  |
| Never preterm delivery | 1 (reference) |  | 1 (reference) |  |
| Ever preterm delivery | 0.04 (-0.03;0.11) | 0.24 | 0.04 (-0.03;0.11) | 0.26 |
| Late preterm delivery | 0.02 (-0.06;0.10) | 0.62 | 0.02 (-0.06;0.10) | 0.67 |
| Very preterm delivery | 0.08 (-0.03;0.19) | 0.15 | 0.08 (-0.03;0.19) | 0.15 |
| **Small for gestational age infant** |  |  |  |  |
| Never small for gestational age infant | 1 (reference) |  | 1 (reference) |  |
| Ever small for gestational age infant | 0.003 (-0.08;0.09) | 0.94 | 0.004 (-0.08;0.09) | 0.93 |
| **Hypertensive disorder of pregnancy** |  |  |  |  |
| Normotensive | 1 (reference) |  | 1 (reference) |  |
| Hypertensive disorder of pregnancy | 0.04 (-0.03;0.12) | 0.24 | 0.06 (-0.01;0.14) | 0.10 |
| Preterm preeclampsia | 0.11 (-0.06;0.27) | 0.20 | 0.12 (-0.04;0.28) | 0.15 |
| Term preeclampsia | 0.06 (-0.04;0.15) | 0.27 | 0.08 (-0.02;0.18) | 0.13 |
| Non- preeclampsia  hypertension | -0.01 (-0.13;0.11) | 0.87 | 0.01 (-0.11;0.13) | 0.88 |
| Results from linear regression multiple imputation analysis. Only NSTEMI cases.  Model I includes adverse pregnancy outcome history; age at first time MI [continuous]  Model II additionally includes diabetes [yes/no]; hypertension [yes/no]; treatment for dyslipidaemia [yes/no]; smoking status [never smoker, current smoker, ex-smoker >1 month]; BMI [continuous].  * BMI: body mass index; CI: confidence interval; MI: myocardial infarction; NSTEMI: non-ST-elevation myocardial infarction; OR: odds ratio. | | | | |

| **Supplemental Table 4.** Association between adverse pregnancy outcome history and high troponin (highest quarter) among women presenting with first time myocardial infarction, only STEMI cases (n=3128) | | | | |
| --- | --- | --- | --- | --- |
|  | **Model I** | | **Model II** | |
|  | OR (95% CI) | *p* | OR (95% CI) | *p* |
| **Preterm delivery**  (n high troponin / n STEMI) |  |  |  |  |
| Never preterm delivery  (1349 / 2644) | 1 (reference) |  | 1 (reference) |  |
| Ever preterm delivery  (239 / 484) | 0.98 (0.80;1.19) | 0.80 | 0.98 (0.81;1.19) | 0.84 |
| Late preterm delivery  (158 / 317) | 1.07 (0.80;1.45) | 0.64 | 1.08 (0.80;1.46) | 0.61 |
| Very preterm delivery  (81 / 167) | 1.05 (0.71;1.57) | 0.80 | 1.09 (0.72;1.61) | 0.71 |
| **Small for gestational age infant**  (n high troponin / n STEMI) |  |  |  |  |
| Never small for gestational age infant  (1380 / 2730) | 1 (reference) |  | 1 (reference) |  |
| Ever small for gestational age infant  (208 / 398) | 1.05 (0.85;1.30) | 0.66 | 1.05 (0.85;1.30) | 0.64 |
| **Hypertensive disorder of pregnancy**  (n high troponin / n STEMI) |  |  |  |  |
| Normotensive  (1391 / 2735) | 1 (reference) |  | 1 (reference) |  |
| Hypertensive disorder of pregnancy  (197 / 393) | 1.02 (0.82;1.26) | 0.87 | 1.03 (0.83;1.28) | 0.80 |
| Preterm preeclampsia  (43 / 85) | 1.09 (0.71;1.69) | 0.69 | 1.12 (0.72;1.73) | 0.63 |
| Term preeclampsia  (96 / 179) | 1.16 (0.86;1.57) | 0.34 | 1.17 (0.86;1.59) | 0.32 |
| Non- preeclampsia  hypertension  (58 / 129) | 0.81 (0.57;1.16) | 0.25 | 0.82 (0.57;1.17) | 0.27 |
| Results from logistic regression multiple imputation analysis. Only STEMI cases.  Model I includes adverse pregnancy outcome history; age at first time MI [continuous]  Model II additionally includes diabetes [yes/no]; hypertension [yes/no]; treatment for dyslipidaemia [yes/no]; smoking status [never smoker, current smoker, ex-smoker >1 month]; BMI [continuous].  * BMI: body mass index; CI: confidence interval; MI: myocardial infarction; OR: odds ratio; STEMI: ST-elevation myocardial infarction. | | | | |

| **Supplemental Table 5.** Association between adverse pregnancy outcome history and high troponin (highest quarter) among women presenting with first time myocardial infarction, only NSTEMI cases (n=5192) | | | | |
| --- | --- | --- | --- | --- |
|  | **Model I** | | **Model II** | |
|  | OR (95% CI) | *p* | OR (95% CI) | *p* |
| **Preterm delivery**  (n high troponin / n NSTEMI) |  |  |  |  |
| Never preterm delivery  (523 / 4375) | 1 (reference) |  | 1 (reference) |  |
| Ever preterm delivery  (97 / 817) | 0.99 (0.79;1.25) | 0.96 | 0.99 (0.78;1.25) | 0.93 |
| Late preterm delivery  (57 / 546) | 0.86 (0.64;1.15) | 0.30 | 0.86 (0.64;1.14) | 0.29 |
| Very preterm delivery  (40 / 271) | 1.28 (0.90;1.81) | 0.17 | 1.28 (0.90;1.81) | 0.17 |
| **Small for gestational age infant**  (n high troponin / n NSTEMI) |  |  |  |  |
| Never small for gestational age infant  (559 / 4703) | 1 (reference) |  | 1 (reference) |  |
| Ever small for gestational age infant  (61 / 489) | 1.06 (0.80;1.40) | 0.70 | 1.06 (0.80;1.40) | 0.70 |
| **Hypertensive disorder of pregnancy**  (n high troponin / n NSTEMI) |  |  |  |  |
| Normotensive  (529 / 4507) | 1 (reference) |  | 1 (reference) |  |
| Hypertensive disorder of pregnancy  (91 / 685) | 1.15 (0.91;1.47) | 0.24 | 1.19 (0.93;1.52) | 0.16 |
| Preterm preeclampsia  (17 / 121) | 1.24 (0.73;2.09) | 0.43 | 1.28 (0.75;2.16) | 0.36 |
| Term preeclampsia  (45 / 339) | 1.15 (0.83;1.60) | 0.39 | 1.19 (0.86;1.66) | 0.29 |
| Non- preeclampsia  hypertension  (29 / 225) | 1.11 (0.75;1.66) | 0.60 | 1.15 (0.77;1.72) | 0.50 |
| Results from logistic regression multiple imputation analysis. Only NSTEMI cases.  Model I includes adverse pregnancy outcome history; age at first time MI [continuous]  Model II additionally includes diabetes [yes/no]; hypertension [yes/no]; treatment for dyslipidaemia [yes/no]; smoking status [never smoker, current smoker, ex-smoker >1 month]; BMI [continuous].  * BMI: body mass index; CI: confidence interval; MI: myocardial infarction; NSTEMI: non-ST-elevation myocardial infarction; OR: odds ratio. | | | | |

| **Supplemental Table 6.** Association between preterm delivery history (normotensive or hypertensive) and STEMI among women presenting with first myocardial infarction (n=8320) | | | | |
| --- | --- | --- | --- | --- |
|  | **Model I** | | **Model II** | |
|  | OR (95% CI) | *p* | OR (95% CI) | *p* |
| **Preterm delivery**  (n STEMI/ n MI) |  |  |  |  |
| Never preterm delivery  (2644 / 7,019) | 1 (reference) |  | 1 (reference) |  |
| Ever preterm delivery  (484 / 1301) | 0.99 (0.87;1.12) | 0.84 | 0.98 (0.87;1.11) | 0.76 |
| Normotensive   preterm delivery  (361 / 985) | 0.96 (0.84;1.10) | 0.57 | 0.92 (0.80;1.06) | 0.23 |
| Hypertensive  preterm delivery  (123 / 316) | 1.08 (0.85;1.36) | 0.54 | 1.22 (0.96;1.54) | 0.11 |
| Results from logistic regression multiple imputation analysis.  Model I includes adverse pregnancy outcome history; age at MI [continuous]  Model II additionally includes diabetes [yes/no]; hypertension [yes/no]; treatment for dyslipidaemia [yes/no]; smoking status [never smoker, current smoker, ex-smoker >1 month]; BMI [continuous].  * BMI: body mass index; CI: confidence interval; MI: myocardial infarction; OR: odds ratio; STEMI: ST-elevation myocardial infarction. | | | | |

| **Supplemental Table 7.** Association between preterm delivery history (normotensive or hypertensive) and invasive revascularization procedure among women presenting with first time myocardial infarction (n=8320) | | | | |
| --- | --- | --- | --- | --- |
|  | **Model I** | | **Model II** | |
|  | OR (95% CI) | *p* | OR (95% CI) | *p* |
| **Preterm delivery**  (n revascularization / n MI) |  |  |  |  |
| Never preterm delivery  (2344 / 7019) | 1 (reference) |  | 1 (reference) |  |
| Ever preterm delivery  (436 / 1301) | 1.01 (0.89;1.15) | 0.82 | 1.01 (0.89;1.15) | 0.91 |
| Normotensive  preterm delivery  (329 / 985) | 1.00 (0.87;1.16) | 0.95 | 0.96 (0.83;1.11) | 0.57 |
| Hypertensive  preterm delivery  (107 / 316) | 1.05 (0.82;1.33) | 0.72 | 1.19 (0.93;1.52) | 0.17 |
| Results from logistic regression multiple imputation analysis.  Model I includes adverse pregnancy outcome history; age at first time MI [continuous]  Model II additionally includes diabetes [yes/no]; hypertension [yes/no]; treatment for dyslipidaemia [yes/no]; smoking status [never smoker, current smoker, ex-smoker >1 month]; BMI [continuous].  * BMI: body mass index; CI: confidence interval; MI: myocardial infarction; OR: odds ratio | | | | |

| **Supplemental Table 8.** Association between preterm delivery history (normotensive or hypertensive) and log z-score troponin among women presenting with first time myocardial infarction (n=8320) | | | | |
| --- | --- | --- | --- | --- |
|  | **Model I** | | **Model II** | |
|  | *β* (95% CI) | *p* | *β* (95% CI) | *p* |
| **Preterm delivery** |  |  |  |  |
| No preterm delivery | 1 (reference) |  | 1 (reference) |  |
| Ever preterm delivery | 0.001 (-0.06;0.06) | 0.98 | -0.001 (-0.06;0.06) | 0.97 |
| Normotensive  preterm delivery | -0.01 (-0.08;0.05) | 0.70 | -0.03 (-0.09;0.04) | 0.44 |
| Hypertensive  preterm delivery | 0.05 (-0.07;0.16) | 0.43 | 0.08 (-0.03;0.20) | 0.17 |
| Results from linear regression multiple imputation analysis.  Model I includes adverse pregnancy outcome history; age at first time MI [continuous]  Model II additionally includes diabetes [yes/no]; hypertension [yes/no]; treatment for dyslipidaemia [yes/no]; smoking status [never smoker, current smoker, ex-smoker >1 month]; BMI [continuous].  * BMI: body mass index; CI: confidence interval; MI: myocardial infarction. | | | | |

| **Supplemental Table 9.** Association between preterm delivery history (normotensive or hypertensive) and high troponin (within the fourth quartile) value among women presenting with first time myocardial infarction (n=8320) | | | | | |
| --- | --- | --- | --- | --- | --- |
|  | **Model I** | | **Model II** | | |
|  | OR (95% CI) | *p* | OR (95% CI) | *p* | |
| **Preterm delivery**  (n high troponin / n MI) |  |  |  |  | |
| No preterm delivery  (1872 / 7019) | 1 (reference) |  | 1 (reference) | |  |
| Ever preterm delivery  (336 / 1301) | 0.97 (0.85;1.11) | 0.70 | 0.97 (0.85;1.11) | 0.67 | |
| Normotensive  preterm delivery  (246 / 985) | 0.92 (0.79;1.08) | 0.31 | 0.90 (0.77;1.05) | 0.18 | |
| Hypertensive  preterm delivery  (90 / 316) | 1.15 (0.89;1.47) | 0.29 | 1.24 (0.96;1.60) | 0.10 | |
| Results from logistic regression multiple imputation analysis.  Model I includes adverse pregnancy outcome history; age at MI [continuous]  Model II additionally includes diabetes [yes/no]; hypertension [yes/no]; treatment for dyslipidaemia [yes/no]; smoking status [never smoker, current smoker, ex-smoker >1 month]; BMI [continuous].  * BMI: body mass index; CI: confidence interval; MI: myocardial infarction; OR: odds ratio. | | | | | |

| **Supplemental Table 10.** Association between hypertensive disorder of pregnancy history and STEMI among women presenting with first myocardial infarction (n=8320), smoking in separate model | | | | | | |
| --- | --- | --- | --- | --- | --- | --- |
|  | **Model I** | | **Model II** |  | **Model III** | |
|  | OR (95% CI) | *p* | OR (95% CI) | *p* | OR (95% CI) | *p* |
| **Hypertensive disorder of pregnancy**  (n STEMI / n MI) |  |  |  |  |  |  |
| Normotensive  (2735 / 7242) | 1 (reference) |  | 1 (reference) |  | 1 (reference) |  |
| Hypertensive disorder of pregnancy  (393 / 1078) | 0.95 (0.83;1.09) | 0.48 | 1.05 (0.91;1.20) | 0.52 | 1.07 (0.94;1.23) | 0.31 |
| Preterm preeclampsia  (85 / 206) | 1.18 (0.89;1.57) | 0.24 | 1.35 (1.02;1.81) | 0.04 | 1.40 (1.05;1.88) | 0.02 |
| Term preeclampsia  (179 / 518) | 0.88 (0.73;1.06) | 0.17 | 0.95 (0.79;1.15) | 0.60 | 0.98 (0.81;1.18) | 0.81 |
| Non-preeclampsia  hypertension  (129 / 354) | 0.95 (0.76;1.18) | 0.63 | 1.03 (0.82;1.29) | 0.79 | 1.06 (0.85;1.33) | 0.61 |
| Results from logistic regression multiple imputation analysis.  Model I includes adverse pregnancy outcome history; age at MI [continuous] Model II additionally includes smoking status [never smoker, current smoker, ex-smoker >1 month]  Model III additionally includes diabetes [yes/no]; hypertension [yes/no]; treatment for dyslipidaemia [yes/no]; BMI [continuous]  * BMI: body mass index; CI: confidence interval; MI: myocardial infarction; OR: odds ratio; STEMI: ST-elevation myocardial infarction. | | | | | | |

| **Supplemental Table 11.** Association between hypertensive disorder of pregnancy history and invasive revascularization procedure among women presenting with first time myocardial infarction (n=8320), smoking in separate model | | | | | | |
| --- | --- | --- | --- | --- | --- | --- |
|  | **Model I** | | **Model II** |  | **Model III** | |
|  | OR (95% CI) | *p* | OR (95% CI) | *p* | OR (95% CI) | *p* |
| **Hypertensive disorder of pregnancy**  (n revascularization / n MI) |  |  |  |  |  |  |
| Normotensive  (2,441 / 7242) | 1 (reference) |  | 1 (reference) |  | 1 (reference) |  |
| Hypertensive disorder of pregnancy  (339 / 1078) | 0.91 (0.79;1.05) | 0.19 | 1.00 (0.87;1.15) | 0.98 | 1.03 (0.89;1.19) | 0.69 |
| Preterm preeclampsia  (77 / 206) | 1.21 (0.90;1.61) | 0.20 | 1.39 (1.04;1.86) | 0.03 | 1.43 (1.07;1.92) | 0.02 |
| Term preeclampsia  (149 / 518) | 0.80 (0.66;0.98) | 0.03 | 0.87 (0.71;1.06) | 0.17 | 0.89 (0.73;1.09) | 0.27 |
| Non-preeclampsia  hypertension  (113 / 354) | 0.92 (0.73;1.16) | 0.50 | 1.01 (0.80;1.28) | 0.93 | 1.04 (0.82;1.31) | 0.76 |
| Results from logistic regression multiple imputation analysis.  Model I includes adverse pregnancy outcome history; age at first time MI [continuous]  Model II additionally includes smoking status [never smoker, current smoker, ex-smoker >1 month]  Model III additionally includes diabetes [yes/no]; hypertension [yes/no]; treatment for dyslipidaemia [yes/no]; BMI [continuous].  * BMI: body mass index; CI: confidence interval; MI: myocardial infarction; OR: odds ratio | | | | | | |
